# Effect of auto-adaptive metal artifact reduction (aMAR) program in cone-beam computed tomography on assessing pre-implant bone levels: Correspondence

**DOI:** 10.34172/japid.026.3671

**Published:** 2026-03-23

**Authors:** Hinpetch Daungsupawong, Viroj Wiwanitkit

**Affiliations:** ^1^Private Academic Consultant, Phonhong, Lao People’s Democratic Republic; ^2^Department of Research Analytics, Saveetha Dental College and Hospitals, Saveetha Institute of Medical and Technical Sciences, Saveetha University, Chennai, India

## Dear Editor,

 This letter concerns the publication on “Effect of auto-adaptive metal artifact reduction (aMAR) program in cone-beam computed tomography on assessing pre-implant bone levels.^1^” The introduction of adaptive metal artifact reduction (aMAR) algorithms in cone-beam computed tomography (CBCT) represents a significant advancement in dental imaging. However, this research lacks a thorough exploration of the specific algorithms and techniques used in aMAR. Although the study by Abesi^1^ describes the use of an aAMR program, the specific construction algorithms and processing steps employed by the software are not described in the Materials and Methods section; their inclusion would enhance methodological transparency and reproducibility.

 A more detailed comparison with existing metal artifact reduction strategies could help improve understanding of the algorithm’s effectiveness.^2,3^ Furthermore, this study could benefit from statistical validation of the algorithm’s performance in a variety of clinical scenarios, as a single technique may not be sufficient in a variety of them. As highlighted in previous clinical evaluations, statistical validation across diverse implant sites and patient populations provides a stronger basis for generalization.^4^ A more comprehensive assessment of the potential of aMAR algorithms could provide a clearer understanding of their advantages over traditional MAR methods.

 One significant disadvantage of aMAR techniques is their reliance on the quality of the first picture capture. If raw CBCT images are noisy or the patient’s posture is not appropriate, even the most complex post-processing features may fail to yield an accurate model image. Furthermore, the performance of aMAR algorithms varies depending on the type of metal present and its spatial arrangement in the photos. Such diversity may restrict their overall relevance in clinical practice. Indeed, systematic reviews have documented that metal type and geometry significantly influence artifact reduction outcomes.^5,6^ Further research is required to assess their robustness in various settings. Future approaches could include using machine learning techniques to improve the real-time adaptation of aMAR algorithms during image collection. Recent studies have demonstrated promising results in reducing dental implant artifacts while adapting to variable anatomical structures.^7^ Using deep learning models, the algorithms may learn from a variety of situations, refining their parameters and eventually providing specific artifact reduction based on each patient’s anatomy and implant site.

 Furthermore, collaboration between doctors and engineers may result in the development of hybrid imaging techniques that combine the strengths of CBCT with other imaging modalities, such as MRI or multi-slice CT, to enhance overall diagnostic capabilities. Hybrid and multimodal imaging have been increasingly recommended to overcome the limitations of single-modality reconstructions.^8^ To increase the novelty, future study could look into integrating augmented reality (AR) with aMAR algorithms. By projecting improved, artifact-free images onto the surgical field, doctors can better visualize bone quality and anatomy during implant placement. Furthermore, innovative data-sharing platforms with a large archive of clinical scenarios could facilitate future algorithm training, allowing doctors to provide real-time feedback on the algorithm’s effectiveness.^9^ Exploring these approaches will broaden not only the potential impact of aMAR algorithms, but also their position in precision dentistry. It should be emphasized that these technical considerations are interpretive and hypothesis-generating and should be validated by dental radiologists and imaging specialists.

## Competing Interests

 The authors declare that they have no competing interests regarding authorship and/or publications of this paper.
